# Acute Fulminant Myocarditis After ChAdOx1 nCoV-19 Vaccine: A Case Report and Literature Review

**DOI:** 10.3389/fcvm.2022.856991

**Published:** 2022-04-21

**Authors:** Chia-Tung Wu, Shy-Chyi Chin, Pao-Hsien Chu

**Affiliations:** ^1^Department of Cardiology, Linkou Medical Center, Chang Gung Memorial Hospital, Taoyuan City, Taiwan; ^2^Department of Medical Imaging and Intervention, Chang Gung Memorial Hospital, Linkou Medical Center and Chang Gung University College of Medicine, Taoyuan City, Taiwan

**Keywords:** COVID-19, vaccine, adenovirus, ChAdOx1, myocarditis

## Abstract

According to recent literatures, myocarditis is an uncommon side effect of mRNA vaccines against COVID-19. On the other hand, myocarditis after adenovirus based vaccine is rarely reported. Here we report a middle-aged healthy female who had acute fulminant perimyocarditis onset 2 days after the first dose of ChAdOx1 vaccine (AstraZeneca) without any other identified etiology. Detailed clinical presentation, serial ECGs, cardiac MRI, and laboratory data were included in the report. Possible mechanisms of acute myocarditis after adenoviral vaccine was reviewed and discussed. To our knowledge, a few cases of myocarditis after Ad26.COV2.S vaccine were reported, and this is the first case report after ChAdOx1 vaccine.

## Introduction

Growing evidence has shown that acute myocarditis is a rare complication after mRNA COVID-19 vaccinations, with an estimated incidence of ∼2 per 100,000 persons after BNT162b2 mRNA vaccine (1, 2), and the risk is higher in adolescent males. Typically, acute myocarditis occurs within 5 days after mRNA vaccination, and the mechanism is still unclear. Myocarditis after adenovirus or protein-based vaccines has seldom been reported. Here, we report the case of a 44-year-old female who had acute fulminant perimyocarditis following the first dose of ChAdOx1 nCoV-19 vaccine with no other identified etiology.

## Case Description

**FIGURE 1:**
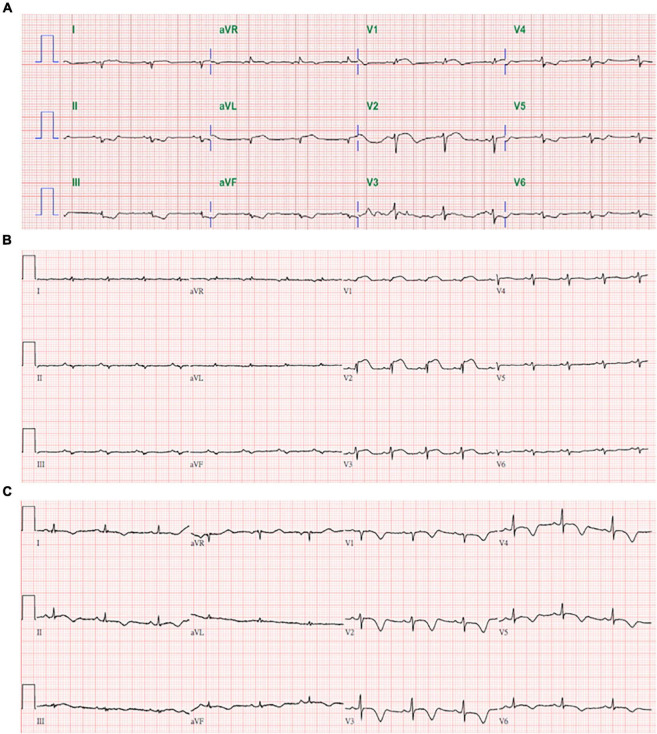
Serial in-hospital ECGs on 8/11 **(A)** from other hospital, 8/13 **(B)**, and 8/24 **(C)**.

A previously healthy 44-year-old Taiwanese female hairdresser (153 cm, 63 kg), without any documented systemic disease, received first dose of ChAdOx1 nCoV-19 vaccine (AstraZeneca) on August 6, 2021. She denied taking any long-term or short -term medication, and had no fever, sore throat, or other symptoms suggesting viral infection within 2 weeks before vaccination. She started to feel persistent stabbing chest pain and breathless approximately 48 h after vaccination. Because the symptoms progressed, she visited the emergency department at another hospital on August 11. Initial troponin I was 17 ng/mL and D-dimer was 1020 ng/mL FEU. ECG showed diffuse low QRS voltage and 1 mm convex ST elevation over V1 and V2 (Figure 1A). Coronary angiography revealed patent coronary arteries, and no pulmonary embolism was found on enhanced CT. She had nausea, vomiting, and abdominal distension after admission. Hypotension developed on August 12, and echocardiography showed poor left ventricular function. Norepinephrine was infused, and she was transferred to our intensive care unit for further management on August 13.

On arrival, her vital signs included temperature 37.2°C, heart rate 108/min, blood pressure 96/77 mmHg (under norepinephrine 0.3 μg/kg/min), respiration 20/min, and O2 saturation 93% under O2 nasal cannula. Fine crackles were heard over bilateral basal lung fields and there was no audible pleural or pericardial friction rub. ECG showed sinus tachycardia, diffuse low QRS voltage, and convex ST elevation over V1 to V3 (Figure 1B). Chest X-way revealed acute pulmonary edema, and echocardiography showed left ventricular diameter 47/39 mm, left ventricular ejection fraction (LVEF) about 35%, and small amount of pericardial effusion. Initial laboratory data on August 13 showed elevated troponin I (8.1 ng/mL), BNP (399 pg/mL), D-dimer (3,815 ng/mL FEU), and ALT (100 U/L). Her creatinine (0.6 mg/dL) and lactate (19 mg/dL) were normal. Complete blood cell count showed leukocytosis (WBC 11,700/μL with segment 88%) with normal hemoglobin (12.4 g/dL) and platelets (251 K/μL). Other relevant in-hospital laboratory results were presented in Supplementary Table 1.

We checked COVID-19, influenza A/B, adenovirus, coxsackievirus, mycoplasma, CMV, EBV, HIV, and markers for autoimmune disease. The results were all negative except for reactive CMV IgG with negative CMV IgM and low C3 66 mg/dL (reference 90∼180). Myocardial biopsy was suggested but she refused. Because D-dimer level increased from 3,815 to 6,433 ng/mL FEU and history of ChAdOx1 vaccination, anti-PF4 antibody level was checked on August 16, and it was 0.15 optical density (normal < 0.4 OD). There was no detectable venous thrombosis by chest CT and peripheral Doppler. Post-vaccine acute fulminant myocarditis is impressed. Since there is no established treatment protocol for post-vaccine myocarditis, we offered the patient standard therapy for heart failure and perimyocarditis.

Initial medication included furosemide, ivabradine, colchicine, and norepinephrine to keep mean arterial pressure above 65 mmHg. After above treatment for 2 days, her appetite and orthopnea gradually improved, and norepinephrine was discontinued on August 16. Her pulmonary edema resolved and troponin I level decreased (daily troponin I 8.1, 6.8, 5.6, 2.1 mg/mL from August 13 to 16). Spironolactone was added and she was transferred to ward on August 18.

**FIGURE 2:**
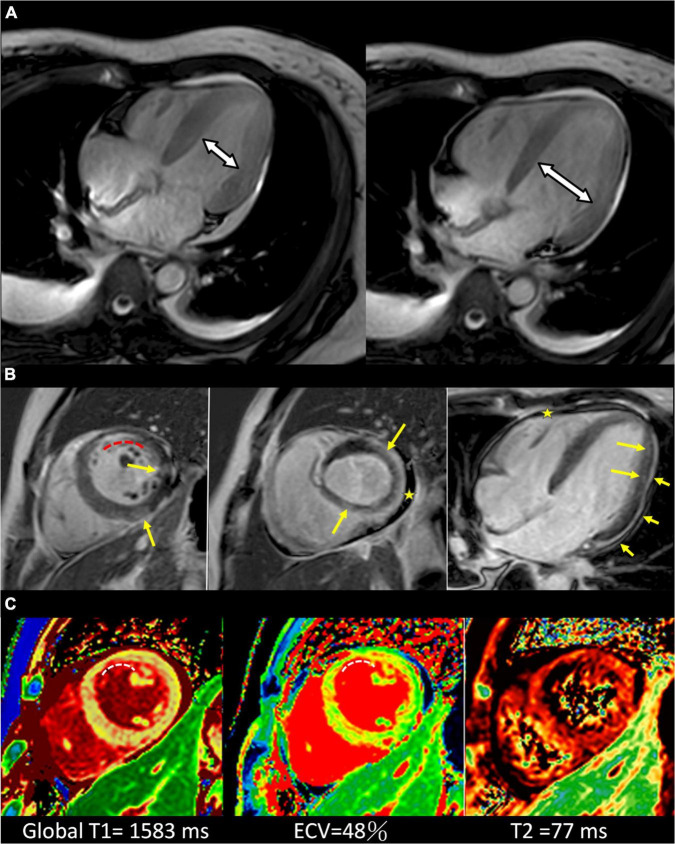
Cardiac MRI of the patient. **(A)** Cardiac MR 4-chamber cine end-systolic (left) and end-diastolic (right) images show the limited LV dimensional change, indicative of the impaired LV systolic function. **(B)** Cardiac MR late Gadolinium enhancement images of short-axis (left, middle) and 4-chamber (right) view. Yellow arrows depict the patchy enhancements sparsely distributed in the mid-layer and subepicardium in a non-ischemic pattern, arrowheads depict pericardial enhancement and stars depict pericardial effusion. The curved dashed line depicts the subendocardial enhancement in the antero-septal subendocardium of LV mid-cavity which is within the LAD territory. **(C)** T1 map (left), ECV map (middle) and T2 map (right) in short-axis views show elevated T1, ECV, and T2 values, indicating acute myocardial injury (global T1 = 1,583 ms, ECV = 48%, T2 = 77 ms; institution-specific cut-off values for abnormal myocardium: T1 global ≥ 1,250 ms, T2 global ≥ 60 ms). CMR findings meet updated 2018 Lake Louise criteria for acute myocarditis (25). The curved dashed lines depict the focally elevated T1 and ECV values in the antero-septal subendocardium, equivalent to the enhanced area depicted in **(B)**.

Cardiac MRI on August 19 showed global LV hypokinesia with LVEF 41.6% and markedly increased LV T1 and T2 signal values (Figure 2). Late Gadolinium enhancement (LGE) images depict the patchy enhancements sparsely distributed in the mid-layer and subepicardium, and subendocardial enhancement in the antero-septal subendocardium of LV mid-cavity. On August 23, her LVEF was 45% by echocardiography and ECG showed evolutionary changes including higher QRS voltage and diffuse T wave inversion (Figure 1C). She was discharged on August 24 with colchicine, losartan, ivabradine, and spironolactone. She had mild dyspnea on exertion and tingling chest pain at discharge, and the symptoms gradually disappeared after discharge. Her latest echocardiography on January 17 2022 showed normal LV diameter (45/31 mm), LVEF 60%, and no pericardial effusion. ECG showed normal sinus rhythm without ST-T changes (Supplementary Figure 3). There was a complete recovery of her fulminant perimyocarditis.

## Discussion

Acute perimyocarditis is an uncommon side effect after vaccination in the pre-COVID-19 era. In the US Vaccine Adverse Event Reporting System (VAERS), total 708 reports met the definition as perimyocarditis from 1990 to 2018 (3). It occurs more commonly in males (79%) than in females, and the most frequently reported vaccines are smallpox (59%), anthrax (23%), and typhoid (13%) vaccines. There is growing evidence that myocarditis is a rare side effect of mRNA vaccines against COVID-19 (1, 2, 4–6). Considering the background incidence of viral myocarditis [about 10–22 per 100,000 individuals per year (7)], a nationwide study in Israel reported a calculated risk ratio of 2.35-fold of acute myocarditis between BNT162b2 (Pfizer) vaccinated and unvaccinated persons (2), and the risk ratio was higher in adolescent males. Most cases of myocarditis occurred within 5 days (median 2 days) following the second dose (1, 2, 8).

While clinical and basic researchers are working on the relationship between myocarditis and mRNA vaccines, myocarditis after adenovirus or protein-based COVID-19 vaccines has seldom been reported. In a recent review of post-COVID-19 vaccination myocarditis (9), only one of the 61 cases received Ad26.COV2.S adenoviral vaccine (Johnson and Johnson) while the other cases all received mRNA vaccine. In another case report of fulminant myocarditis after Ad26.COV2.S vaccine, the patient expired within 24 h despite of ECMO support (10). Autopsy revealed lymphohistocytic myocarditis. In our report, because the patient refused myocardial biopsy, the diagnosis of myocarditis is based on diagnostic criteria from European Society of Cardiology Working Group on Myocardial and Pericardial Diseases (Table 1) (11). All diagnostic criteria include abnormal ECG and echocardiography, elevated Troponin I, and myocardial damage by cardiac MRI were met and coronary angiography showed patent coronary arteries. Because her symptoms onset 2 days after the first dose of ChAdOx1 nCoV-19 vaccine without any other identified etiology, vaccine-related myocarditis was highly suspected. Currently there is no established test to confirm the causal relationship. According to the report from VAERS, rates of post-vaccine myocarditis for females aged 40–49 years was 0.1/1.1 per 1 million doses after first/second dose of BNT162b2 and 0.2/1.4 after first/second dose of mRNA-1273 vaccine (Moderna) (12). The reported incidence of myocarditis after mRNA vaccines is quite low at her age as well. A phase 3 study of ChAdOx1 nCoV-19 vaccine enrolled 32,451 participants, and the number was still underpowered to detect uncommon side effects such as vaccine-induced immune thrombotic thrombocytopenia (VITT). Although no myocarditis was reported in either group, two cases with cardiac disorders were reported as medically attended adverse events in the ChAdOx1 group compared to 0 events in the placebo group (13).

**TABLE 1:** Diagnostic criteria for clinically suspected myocarditis from European society of cardiology working group on myocardial and pericardial diseases (11).

Clinical presentations
1. Acute chest pain, pericarditic, or pseudo-ischemic.
2. New-onset (days up to 3 months) or worsening of: dyspnea at rest or exercise, and/or fatigue, with or without left and/or right heart failure signs
3. Sub-acute/chronic (> 3 months) or worsening of: dyspnea at rest or exercise, and/or fatigue, with or without left and/or right heart failure signs
4.Palpitation, and/or unexplained arrhythmia symptoms and/or syncope, and/or aborted sudden cardiac death
5. Unexplained cardiogenic shock

**Diagnostic criteria**

1. ECG/Holter/stress test: Newly abnormal 12 lead ECG and/or Holter and/or stress testing, any of the following: I to III degree atrioventricular block, or bundle branch block, ST/T wave change (ST elevation or non ST elevation, T wave inversion), sinus arrest, ventricular tachycardia or fibrillation and asystole, atrial fibrillation, reduced R wave height, intraventricular conduction delay (widened QRS complex), abnormal Q waves, low voltage, frequent premature beats, supraventricular tachycardia.
2. Myocardiocytolysis markers: Elevated TnT/TnI
3. Functional and structural abnormalities on cardiac imaging (Echo/Angio/CMR): New, otherwise unexplained LV and/or RV structure and function abnormality (including incidental finding in apparently asymptomatic subjects): regional wall motion or global systolic or diastolic function abnormality, with or without ventricular dilatation, with or without increased wall thickness, with or without pericardial effusion, with or without endocavitary thrombi
4. Tissue characterization by CMR: Edema and/or LGE of classical myocarditic pattern

*Clinically suspected myocarditis if ≥ 1 clinical presentation and ≥ 1 diagnostic criteria from different categories, in the absence of: (1) angiographically detectable coronary artery disease (coronarystenosis ≥ 50%); (2) known pre-existing cardiovascular disease or extra-cardiac causes that could explain the syndrome (e.g., valve disease, congenital heart disease, hyperthyroidism, etc.). Suspicion is higher with higher number of fulfilled criteria.*

*If the patient is asymptomatic ≥ 2 diagnostic criteria should be met.*

Our patient had negative anti-PF4 antibody, so the pathophysiology was different from VITT. There are several possible mechanisms that may lead to myocarditis after ChAdOx1 vaccination. First, adenovirus is an established cause of acute myocarditis (14). Adenovirus can enter cardiomyocytes by binding to a common transmembrane receptor [coxsackievirus and adenovirus receptor (CAR)], induce direct myocardial injury, and trigger an uncontrolled immune response even after viral clearance (15). The genes of dsDNA adenovirus are classified into early genes (E 1–4) which encode proteins for DNA replication and late genes (L 1–5) which encode structural proteins. The viral vector of ChAdOx1 vaccine is a chimpanzee adenovirus (ChAd), which can evade pre-existing human immunity. The ChAd was vectorized by deleting E1/E3 and modifying E4 to reduce virulence and replication in human body (16). In an animal study on rhesus macaques, virus replication in the respiratory tract was limited after vaccination with ChAdOx1 (17). This may explain why a throat swab for adenoviral antigen was negative in our patient.

Another potential mechanism is the molecular similarity between SARS-CoV-2 spike protein and human antigens. Commercially available mouse monoclonal antibodies against SARS-CoV-2 spike protein have been shown to cross-react with some human protein sequences, including α-myosin and actin (18). Repeated antigen exposure may also trigger a dysregulated host response in certain individuals, resulting in polyclonal B-cell expansion, immune complex formation, and inflammation. Induction of anti-idiotype antibodies (antibody 2 against antibody 1) is another possible mechanism for myocarditis after SARS-CoV-2 infection or vaccination (19). Post-vaccination myocarditis bears some similarities to anti-idiotype antibody related myocarditis after viral infections (20). These autoimmune hypotheses can explain the higher incidence of myocarditis after second dose comparing to first dose.

The cardiac MRI in our patient showed increased LV T1 and T2 signal values, indicating acute myocardial injury. Patchy enhancements in the mid-layer and subepicardium by LGE can be observed in the infarction-caused fibrosis and also myocardial damage/necrosis such as myocarditis. These changes are similar to the finding from other myocarditis cases after mRNA vaccination (9). An unusual finding is the enhancement in the antero-septal subendocardium of LV by LGE image, and the pattern is compatible with myocardial infarction with non-obstructive coronary arteries (MINOCA) (21). Common causes of MINOCA are coronary dissection, coronary artery or microvascular spasm, Takotsubo cardiomyopathy, and myocarditis (22). The MRI abnormalities may be related to the degree of myocardial damage, but cannot explain the etiology. Clinically, most cases of myocarditis following mRNA vaccination have been reported to be mild. In a report from Israel, 41 of the 54 cases were mild, one case received ECMO support, and one case died of unknown cause after discharge (1).

According to Australian Guidance on Myocarditis and Pericarditis after mRNA COVID-19 Vaccines (23), initial evaluation of post-vaccine myocarditis/pericarditis was similar to that of typical myocarditis, including history taking, 12-lead ECG, chest X-ray, and Troponin level. Suspected cases require referral to a cardiologist for further investigations including echocardiogram, coronary angiography, and cardiac MRI. Endomyocardial biopsy is rarely indicated, as determined by cardiologist. Often supportive treatment is all that is required. Another important issue is about the subsequent COVID-19 vaccines after post-vaccine myocarditis. According to a recent report about the risk of a second COVID-19 vaccine in 40 patients with VITT after first dose of ChAdOx1 nCoV-19 vaccine (5 patients received ChAdOx1 nCoV-19 again, 2 received mRNA-1273, and 33 received BNT162b2), none of the 40 patients had relapse of symptoms or severe adverse reactions (24). To date, there is no published report about the risk of subsequent vaccine on patients with post-vaccine myocarditis. The Canadian National Advisory Committee on Immunization recommends that individuals who had myocarditis/pericarditis after a first dose of mRNA vaccine should wait to receive a second dose until more information is available. In our case, the patient decided to postpone the schedule of second vaccine.

## Conclusion

Acute pericarditis/myocarditis is a rare but existing side effect after mRNA COVID-19 vaccination, and the incidence is higher among young and adolescence males. Our report demonstrated the possibility of acute myocarditis after ChAdOx1 nCoV-19 vaccine, and the pathophysiology is different from VITT. The risk of post-vaccine myocarditis has affected the public policy in some countries. For example, Finland and Sweden have limited the use of mRNA-1273 vaccine in young people since October 2021. Although myocarditis is potentially lethal, benefits of COVID-19 vaccination (9) still far outweigh this uncommon side effect. Without appropriate evidences, policies about vaccine should be made carefully. Further information about the mechanism and long-term clinical outcome of post-vaccine myocarditis is needed for physicians to manage and give advice about subsequent vaccination on these affected individuals.

## Data Availability Statement

The original contributions presented in the study are included in the article/Supplementary Material, further inquiries can be directed to the corresponding author/s.

## Ethics Statement

Written informed consent was obtained from the individual(s) for the publication of any potentially identifiable images or data included in this article.

## Author Contributions

C-TW took care of the patient and wrote the report. S-CC performed cardiac MRI and provided the image. P-HC revised the report. All authors contributed to the article and approved the submitted version.

## Conflict of Interest

The authors declare that the research was conducted in the absence of any commercial or financial relationships that could be construed as a potential conflict of interest.

## Publisher’s Note

All claims expressed in this article are solely those of the authors and do not necessarily represent those of their affiliated organizations, or those of the publisher, the editors and the reviewers. Any product that may be evaluated in this article, or claim that may be made by its manufacturer, is not guaranteed or endorsed by the publisher.
