# Supplementary material for: Acute Fulminant Myocarditis After ChAdOx1 nCoV-19 Vaccine: A Case Report and Literature Review
Source: Front Cardiovasc Med. 2022 Apr 21;9:856991. doi: 10.3389/fcvm.2022.856991 (PMC9068965; doi:10.3389/fcvm.2022.856991)

Supplementary Material

Table 1. Relevant in-hospital laboratory data

	Reference	8/13	8/14	8/15	8/16	8/18	8/23
**Troponin I (ng/mL)**	<0.3	8.1	6.8	5.6	2.1		
**Total CK (U/L)**	Female 20-180	392	301				
**CK-MB (%)**	0-4	7.2	6.1				
**BNP (pg/mL)**	<100	399					
**Lactate (mg/dL)**	4.5-19.8	20.6	17.3				
**CRP (mg/L)**	<5	9			51.6*	66.9	5.9
**Na (mEq/L)**	136~146	137		136	134	134	138
**K (mEq/L)**	3.5~5.1	4.3	3.9	3.6	4.3	3.5	4.1
**Ca (mg/dL)**	8.6~10.3	7.4	7.9	7.5	7.5		
**Mg (mEq/L)**	1.3-2.1	1.6	2.0	2.0	2.0		
**BUN (mg/dL)**	7~25	14.8			7.7	10.9	13
**Creatinine (mg/dL)**	Female 0.44~1.03	0.56			0.46	0.53	0.49
**ALT (U/L)**	≦36	100	149				55
**ESR (mm/hr)**	0-20	2					
**PT INR**	≦1.2	1.2					
**D-Dimer (FEU ng/mL)**	≦550	3815	6433				
**Fibrinogen (mg/dL)**	190-380		175				
**Hemoglobin (g/dL)**	Female 12.0~16.0	12.4		10.6	10.5	10.7	11.6
**Platelets (1000/uL)**	150-400	251		237		271	337
**WBC (1000/uL)**	3.5 ~11.0	11.7		11.8		9.9	7.2
**Segment (%)**	42-74	88.2		84		78.2	69.3
**Lymphocyte (%)**	20-56	8.8		10.4		14.6	20.3
**Monocyte**	0-12	2.6		5		4.6	6.6
**Eosinophil**	0-5	0.1		0.3		2.2	3.5
**Basophil**	0-1	0.3		0.3		0.4	0.3

*Urinary tract infection during admission

Table 2. In-hospital medication

<table>
<tr><th>Medication</th><th>Aug. 13</th><th>14</th><th>15</th><th>16</th><th>17</th><th>18</th><th>19</th><th>20</th><th>21</th><th>22</th><th>23</th></tr>
<tr><td>Norepinephrine</td><td colspan="4">Keep MAP > 65 mmHg</td><td></td><td></td><td></td><td></td><td></td><td></td><td></td></tr>
<tr><td>Furosemide</td><td colspan="4">IV 20 mg BID</td><td colspan="4">Oral 20 mg QD</td><td></td><td></td><td></td></tr>
<tr><td>Ivabradine</td><td></td><td></td><td colspan="3">2.5 mg BID</td><td colspan="6">5 mg BID</td></tr>
<tr><td>Colchicine</td><td colspan="11">0.5 mg QD</td></tr>
<tr><td>Spironolactone</td><td></td><td></td><td></td><td></td><td></td><td colspan="6">25 mg QD</td></tr>
<tr><td>Losartan</td><td></td><td></td><td></td><td></td><td></td><td colspan="6">25 mg QD</td></tr>
<tr><td>Ceftriaxone *</td><td></td><td></td><td></td><td colspan="5">IV 1g Q12h</td><td></td><td></td><td></td></tr>
</table>

*For urinary tract infection

Figure 1. Chest X-ray on 8/13/2021 (left) and 8/30/2021 (right)

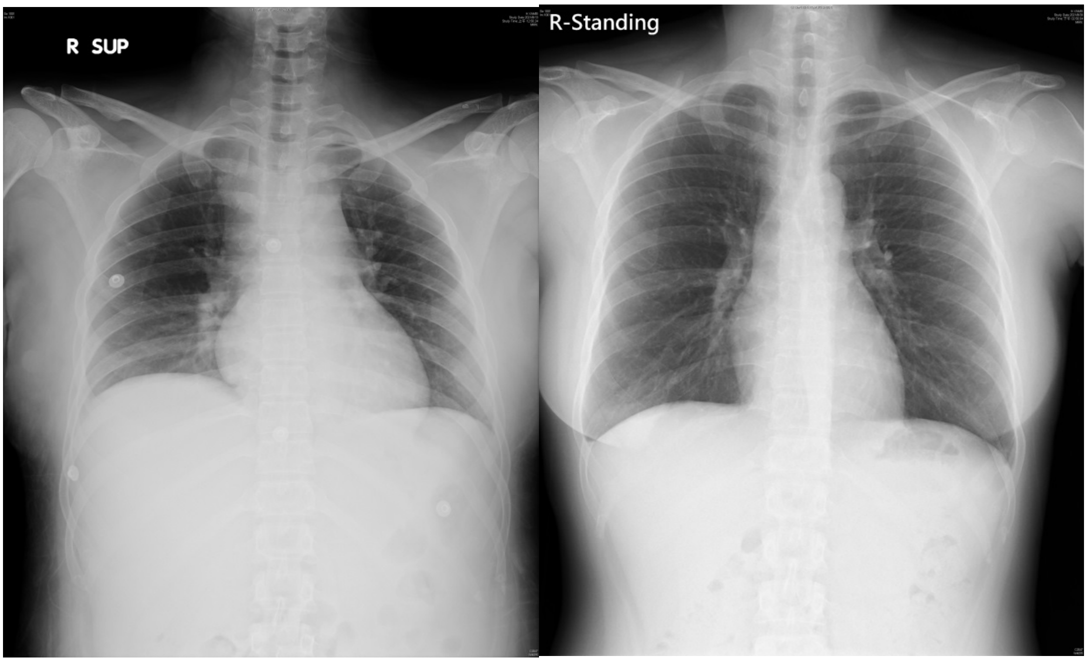

Figure 2. Parasternal long axis view of echocardiogram on 8/13/2021

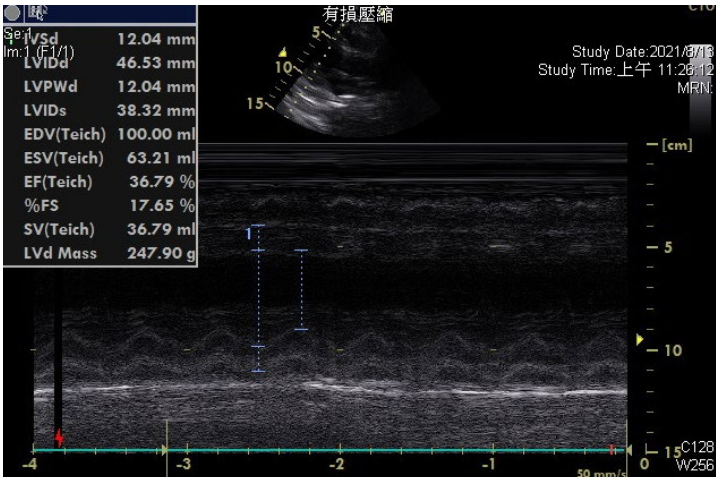

Figure 3. ECG and parasternal long axis view of echocardiogram on 1/17/2022

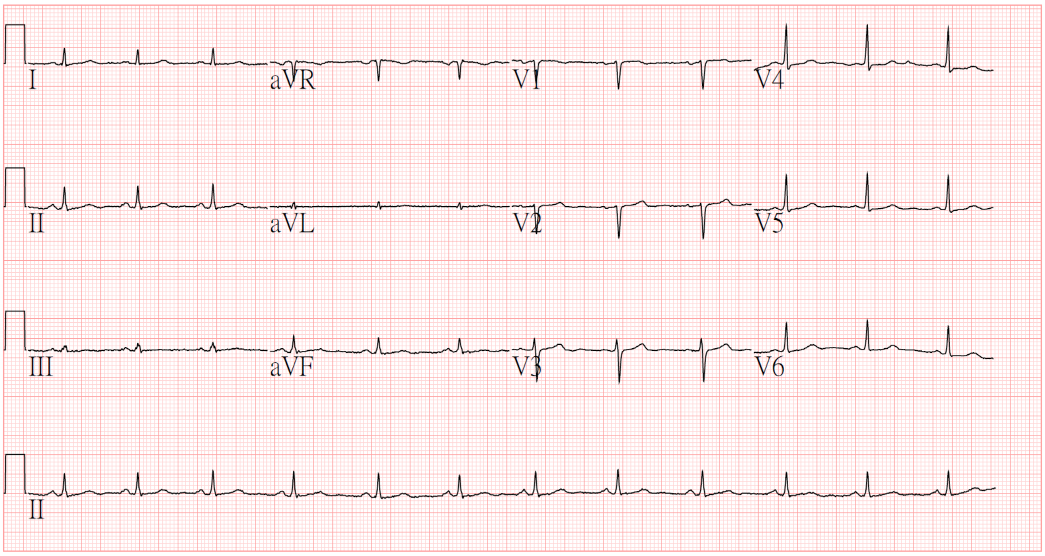

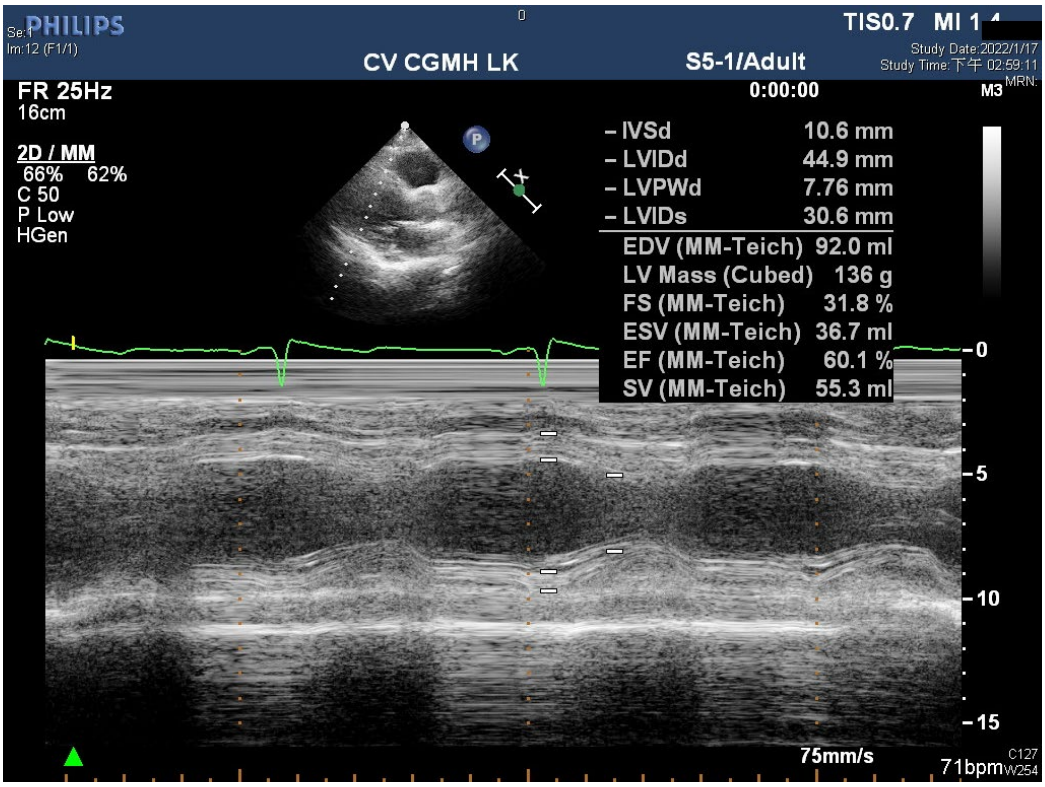

Supplement: Supplementary file 1 [file Data_Sheet_1.pdf]
